# The Chinese herbal formula Huoxiang Zhengqi for atopic dermatitis with dampness pattern (CHARM): a study protocol for a double-blinded randomized controlled trial

**DOI:** 10.1186/s13063-020-05014-6

**Published:** 2021-01-19

**Authors:** Meiling Xuan, Xiaohui Guo, Hongyi Li, Ting Xie, Xiumei Mo, Zehuai Wen

**Affiliations:** 1 Key Unit of Methodology in Clinical Research, Guangdong Provincial Hospital of Chinese Medicine, 111 Dade Road, Guangzhou, 510120 China; 2 State Key Laboratory of Dampness Syndrome of Chinese Medicine, Second Affiliated Hospital of Guangzhou University of Chinese Medicine, Guangzhou, 510120 China; 3 Department of Dermatology, Guangdong Provincial Hospital of Chinese Medicine, 111 Dade Road, Guangzhou, 510120 China

**Keywords:** Atopic dermatitis, Eczema, Randomized controlled trial, Huoxiang Zhengqi, Chinese medicine

## Abstract

**Background:**

Atopic dermatitis (AD) is a complex, common inflammatory skin disease. The Chinese herbal formula Huoxiang Zhengqi (HXZQ) has been a common dermatosis treatment in China for many years, but there is no high-level evidence for its effect on AD/eczema. The aim of this trial is to examine the efficacy and safety of HXZQ treating AD/eczema patients.

**Methods:**

This is a double-blind, multi-center, randomized controlled trial comparing HXZQ to a placebo. It will consist of 4 weeks’ treatment and 4 weeks of follow-up. A total of 218 participants will be randomly allocated into two groups—an HXZQ group and a placebo group, from 7 hospitals in China. Patients diagnosed with AD will be enrolled if they are in accordance with CM dampness pattern, have body surface area (BSA) of 1–10%, have investigator’s global assessment (IGA) of 1–3, have age between 18 and 70 years, and provide signed informed consent. The Eczema Area and Severity Index (EASI) is the primary outcome. The secondary outcomes are the numerical itch rating scale, IGA, BSA, Skindex-29, and EQ-5D-5L score, from baseline to the end of the treatment. Analysis will be on intention-to-treat and per-protocol subject analysis principles.

**Discussion:**

The goal of this trial is to evaluate the efficacy and availability of HXZQ oral liquid in treating AD/eczema in terms of symptoms and eczematous lesions. It will also address whether it has positive effect on QoL.

**Trial registration:**

Chinese Clinical Trial Registry (http://www.chictr.org.cn/index.aspx): Chinese herbal formula Huoxiang Zhengqi for atopic dermatitis with dampness pattern (CHARM): a double-blinded randomized controlled trial, ChiCTR1900026700. Registered on 19 October 2019.

## Background

Atopic dermatitis (AD) is a chronic inflammatory skin disorder characterized by intense itching and eczematous lesions. It diminishes quality of life and incurs healthcare costs. AD prevalence has increased in recent decades in China, varying between 0.70% (among children) and 8.3% (among teenagers) [1]. A recent survey has reported that the prevalence of AD among adults was 4.6%, nearing rates for childhood [2].

Moisturizing and basic care are the cornerstone of AD management. A systematic review has shown that moisturizers prolong time to flare-up and reduce topical corticosteroid (TCS) use, thus mitigating the effects of atopic eczema [3]. Topical daily use of moisturizers has also been shown to be tolerated by patients, and to improve treatment of mild to moderate adulthood AD [4]. It has been established that systemic therapies are options for patients with moderate-to-severe AD for whom first-line therapies are ineffective [5]. However, there are limited options for prescription topical therapy for AD. TCSs are used in the management of AD and are the mainstay of anti-inflammatory therapy. In addition, some side effects, such as skin atrophy, will resolve after discontinuing TCS use, but this may take months. Given the recommendations from the guidelines, continuous application of TCS over extended durations should be avoided [6]. Chinese herbal medicine (CHM) has been a common treatment for symptoms of AD in China for centuries. Findings from a systematic review have suggested that Chinese herbal medicine mitigates AD and does not cause severe adverse events [7].

The Chinese herbal formula Huoxiang Zhengqi (HXZQ) is a classical prescription. It was documented by the Prescriptions People’s Welfare Pharmacy (Taiping Huimin Hejiju Fang), a proprietary Chinese medicine standard compiled by the Song Dynasty government in the early twelfth century. It is a dampness-dispelling formula composed of *Pericarpium Arecae* (Dafupi), *Radix Angelicae Dahuricae* (Baizhi), *Perillae* (Zisu), *Poria* (Fuling), *Rhizoma Pinelliae* (Banxia), *Rhizoma Atractylodis Macrocephalae* (Baizhu), *Pericarpium Citri Reticulatae* (Chenpi), *Cortex Magnoliae Officinalis* (Houpu), *Radix Platycodonis* (Jigeng), *Herba Pogostemonis* (Huoxiang), and *Radix Glycyrrhizae* (Gancao). It has been used to treat various diseases (gastrointestinal cold and acute gastroenteritis) associated with the dampness pattern (*Shi Zheng*) in Chinese medicine (CM). It has had an exceptional safety profile over hundreds of years [8]. At present, it is an over-the-counter (OTC) medication recorded in the Chinese Pharmacopoeia 2015 edition, and it comes in many dosage forms, such as capsules, boluses, pills, and liquids [9]. Pharmacological studies have shown that HXZQ and its pharmaceutical ingredients have antispasmodic, antibacterial, antiallergic, immunity-enhancing effects, both in vivo and vitro [10–13]. HXZQ liquid has obvious inhibitory effect on *Staphylococcus aureus* in vitro. HXZQ liquid drug-serum may neutralize and block IgE and has inhibitive effects on degranulation of cells when resensitized by antigens. Besides, it can block allergic response type I [14]. There is positive effect of HXZQ on immune and metabolic functions, intestinal flora disturbance of rat model with the dampness pattern (*Shi Zheng*) in CM [15–17]. Thus far, it is shown that *Staphylococcus aureus* colonization is a characteristic of skin lesions and drives inflammation in AD [18–20]. In the composition of HXZQ, most of the pogostone (main components of *Herba Pogostemonis*) and its derivatives, *Perillae* extract, *Cortex Magnoliae Officinalis,* Hesperidin (the main active composition of *Pericarpium Citri Reticulatae*) possess different levels of antibiotic activities, in which mainly against *Staphylococcus aureus* [21–26]. Since being on the market, it also has a good safety record [8]. In recent years, several case reports and observational studies have reported that oral administration and external use of HXZQ formula can relieve itching and lesions in AD and eczema adult and child patients [27–31].

AD is the most commonly used term for the condition it describes; eczema is another term often used by patients and physicians alike [32]. However, the nomenclature for AD remains controversial. In China, AD and eczema have two separate sets of guidelines. The diagnostic criteria in the AD guidelines are based on the presence of several essential and associated features, which are recommended by the Williams diagnostic criteria. Eczema diagnosis relies primarily on clinical presentation, eczematous lesions such as erythema, papules, blisters, lichenification, infiltration, and pruritus. In CM theory, dampness is a dominant syndrome in AD/eczema’s pathogenesis and disease progression. Previous clinical practice on diseases with the dampness pattern, such as AD/eczema, has shown some improvement in symptoms. However, to date, there is no evidence based on research using a placebo as control. This multi-center, randomized double-blind, placebo-controlled study is designed to evaluate the efficacy and safety of HXZQ in adults with AD/eczema in China.

## Methods/design

### Design and setting

**Fig. 1:**
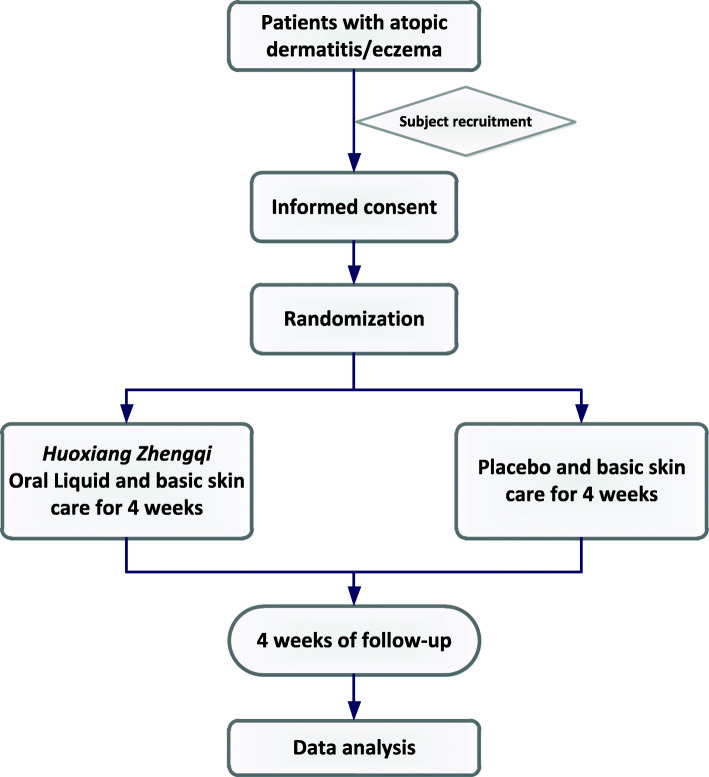
Study flow chart

This is a multi-center, double-blinded, randomized controlled trial (RCT), which will be conducted in 7 third-grade first-class hospitals in China. The study aims to enroll 218 patients with eczema over a 3-year period. Patients will be randomized at a ratio of 1:1 to receive either HXZQ oral liquid (20 ml twice daily) or a placebo. A 4-week follow-up period will continue until the study ends. This trial was approved by the seven sites’ ethics committees and required archival filing management with six other sites’ ethics committees. A flow diagram of the trial is shown in Fig. 1, and the schedule of enrolment, intervention, and assessments is presented in Table 1.

**Table 1:**
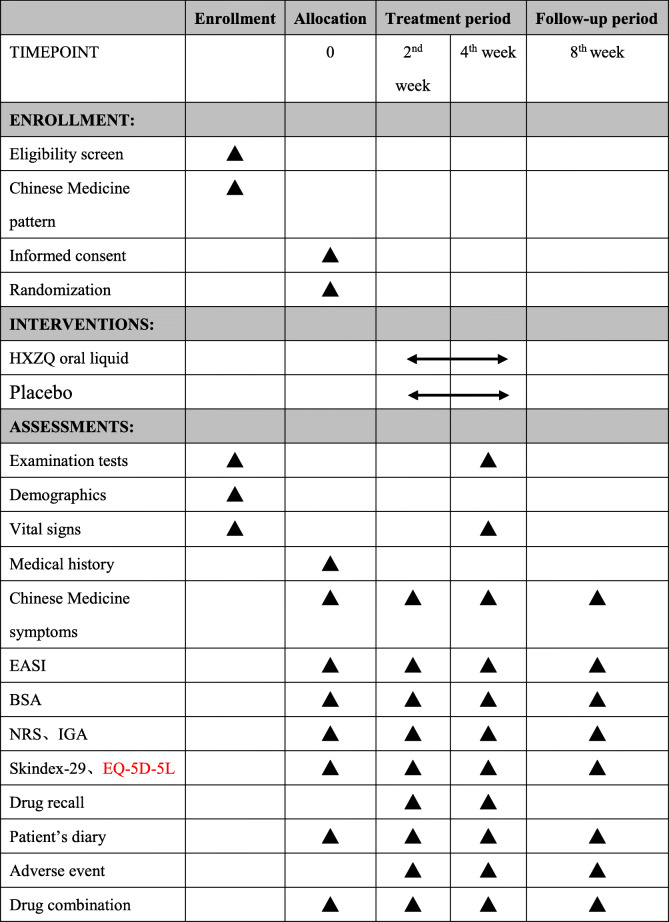
Schedule of enrollment, treatment, and assessment

### Participants

#### Recruitment

Participants will be recruited through social media, advertisements in local newspapers, or posters in hospitals. Some participants may spontaneously contact research centers.

#### Inclusion criteria

Patients will be enrolled if they have been diagnosed with eczema, and in accordance with the CM pattern criteria for dampness. They must be between 18 and 70 years of age, have 1% < body surface area (BSA) < 10%, have 1 ≤ investigator’s global assessment (IGA) ≤ 3, and provide signed informed consent.

#### Exclusion criteria

1. Those with any uncontrolled cardiovascular, respiratory, digestive, urinary, or hematological disease; known cancer or mental illness
2. Those who have ever used HXZQ for treating eczema
3. Anyone allergic to any medicine or ingredients used in the study
4. Those whose examination results show alanine aminotransferase (ALT) or aspartate aminotransferase (AST) levels exceeding 2 times the upper limit normal value; or total bilirubin or blood urea nitrogen (BUN) level exceeds 1.5 times the upper limit normal value during the screening period
5. Women who are pregnant or lactating or who plan to become pregnant within 3 months
6. Medication used: (1) systemic therapy with immunomodulatory or immunosuppressive agents (including *Tripterygium wilfordii*) within the previous 4 weeks; (2) topical or systematic therapy with antibiotics or antihistamines within the previous 2 weeks; (3) phototherapy (such as PUVA, UVA, or UVB) within the previous 4 weeks; (4) topical therapy with immunomodulator within the previous 2 weeks; or (5) topical or systematic use of any over-the-counter drugs within the previous week
7. Those who are participating in any other clinical trial or had participated in a clinical trial within the previous month
8. Those who are considered unfit for the trial

#### CM pattern differentiation criteria

The CM pattern diagnostic criteria will follow the Criteria of Diagnosis and Therapeutic Effect and Syndromes in Traditional Chinese Medicine (issued by the National Administration of Traditional Chinese Medicine, China) and the Textbook of Diagnostics of Traditional Chinese Medicine [33]. CM pattern will be judged by senior deputy chief physician during screening period. The dampness pattern should meet two major symptoms or one major symptom with one secondary symptom.

- Major symptoms: mental fatigue, anorexia, loose stool or watery diarrhea, white sticky greasy tongue coating
- Secondary symptoms: heavy sensation in limbs, abdominal distension, pale tongue or pale teeth-marked tongue, soggy pulse or moderate pulse

### Interventions

Patients in the experimental group will receive HXZQ oral liquid, 20 ml twice daily, for 4 weeks. Placebo liquid will be given to patients in the control group 20 ml twice daily, for 4 weeks. The placebo will consist of glycyrrhizin, bitterant, caramel color, and trace amounts of cinnamon extract and ginger juice. It will be consistent with the HXZQ oral liquid in appearance, taste, and weight, to the greatest extent possible. The HXZQ and placebo oral liquids will be manufactured by Taiji Group Chongqing Fuling Pharmaceutical Co. Ltd. (Chongqing, China) according to the requirements of the good manufacturing practice, and will be met the hygienic requirements of the oral liquid product.

#### Rescue and concomitant treatment

Routine treatment, such as urea ointment, is recommended for each group, even on nonlesional skin, based on doctors’ recommendations. In cases of serious itching, cetirizine hydrochloride or levocetirizine is to be used as a rescue drug, and dosage and frequency are to be recorded for each use.

#### Randomization and blinding

Eligible patients will be enrolled at each site. The participants will be randomly allocated to either the HXZQ group or the placebo group at a 1:1 ratio through an interactive web response system (IWRS). A computer-generated random list for center-stratified method and permuted block size will be used in IWRS and performed by the Institute of Basic Research in Clinical Medicine (IBRCM), China Academy of Chinese Medical Science with SAS 9.2 (SAS Institute Inc., Cary, USA).

The randomization results and blinding codes will be kept strictly confidential; they will be concealed until interventions are all assigned, and enrollment, follow-up, data collection, data cleaning, and analysis are complete. Participants and researchers, including paramedics, investigators, outcomes assessors, and statisticians, will be unaware of the allocation.

### Outcome measures

The primary outcome is change in the Eczema Area and Severity Index (EASI) scores from baseline to the end of the 4th week. The EASI is a composite index recommended by the global Harmonising Outcome Measures for Eczema (HOME) as the core outcome measurement instrument for AD [34]. It includes an assessment of disease extent and body surface area, converted to a proportional factor (on a scale of 0–6), for four body regions (head/neck, upper limbs, trunk and lower limbs). In this trial, the proportions are 10% for head and neck, 20% for upper extremities, 30% for trunk, and 40% for lower extremities. It also includes an assessment of erythema, infiltration and/or papulation, excoriation, and lichenification (on a scale of 0–3 for each one). The total EASI score is the sum of these four body-region scores and the range from 0 to 72 [35].

Secondary outcome measurements are the change in the pruritus numerical rating scale (NRS) of itch, IGA, BSA, Skindex-29, and EQ-5D-5L score from baseline to the end of the treatment period.

The pruritus NRS is a scale from 0 to 10, which assess the pruritus intensity (0 = no itch and 10 = worst itch imaginable). IGA scale uses clinical characteristics to assess overall appearance of the lesions at a given time point (0—clear; 1—almost clear; 2—mild, but noticeable; 3—moderate; 4—severe; 5—very severe). BSA refers the total surface area of the human body. BSA of lesional involvement in-of-itself is also an important aspect of AD severity. Hand surface area (HSA) will be used to guide the assessment of involved surface area. HAS represents 1% of the total body surface area [36]. Skindex-29 is one of the best dermatological instruments for measuring dermatology-specific quality of life (QoL). It is a 30-item dermatology-specific QoL instrument for adults with an unscored item no.18, measuring 3 domains—emotions, functioning, and symptoms. Each item is rated on a 5-point Likert scale (never, rarely, sometimes, often, all the time), with higher scores indicating worse health status. And the EQ-5D is a generic instrument for describing and valuing health. It defines health in terms of 5 dimensions: mobility, self-care, usual activities, pain/discomfort, and anxiety/depression [37]. The 5-level version of the EQ-5D (EQ-5D-5L) will be used as secondary outcome in this trial.

EASI50 and EASI75 are regarded as secondary outcomes as well. EASI50 and EASI75 refer to an EASI score showing improvement exceeding 50% and 75%, respectively, during the treatment period [13]. Antihistamine use will be recorded at baseline, week 2, week 4, and week 8. CM syndrome and tongue and pulse condition will be assessed at the beginning and at the end of the study.

### Safety assessment

Adverse events (AEs) that occur during this trial will be recorded and reported to investigators, and the causality between AE and intervention will be assessed according to the WHO Uppsala Monitoring Centre System for Standardized Case Causality Assessment [38]. An independent data and safety monitoring committee will assess any safety data requested during the trial. Investigators will address AEs using the IWRS in a timely manner in cases of emergency. They will apply to break the blindness if necessary.

### Sample size calculation

Based on Belloni et al.’s study [39], MAS063D (Atopiclair) used to manage mild to moderate AD can achieve a 4 point improvement in EASI score over a 22-day period. A systematic review showed that EASI score in the oat moisturizer group was 4.38 points lower than that of the vehicle or no treatment groups [3]. The minimal important difference (MID) of EASI is 3.4, and it is hypothesized that EASI change in HXZQ oral liquid therapy for AD/eczema from baseline is 4.8 less than placebo. According to this hypothesis, and as calculated by PASS 11.0 (NCSS, LLC, Kaysville, Utah, USA), a sample size of 92 for each group can achieve 90% power and thus rule out a two-sided type I error of 5%. In this way, it can detect a superiority margin difference of 4.8 (standard deviation, SD = 3) for this two-arm trial with equal allocation in each group. Considering a 15% loss to follow-up, the total sample size should be adjusted to 218.

### Data management and quality control

Case report forms and information on the NRS, Skindex-29, and EQ-5D-5L scales will be collected in this trial. All data will be entered into the electronic data capture system after the subject completes the last visit at each center.

To ensure the trial process complies with the trial protocol, physicians, assessors, and research assistants will attend a training workshop before it commences. All investigators and study assistants will also be provided with written protocol documents. The data from all of the sites will be checked regularly by researchers and overseen by monitors. The trial’s monitoring tasks will be entrusted to Beijing Yaohai Ningkang Pharmaceutical Technology Co. Ltd. The auditing and inspection of the trial will be performed by the department of science research at the Guangdong Provincial Hospital of Chinese Medicine and the Office of the National Key Research and Development Program of China. The data monitoring committee will assess the safety data and the critical efficacy outcomes.

A transportation allowance will be provided to the participants for return visit. The disease severity evaluation and QoL measurement take approximately 15–30 min. Participants can withdraw from this trial at any time for any reason. Investigators should ask if the patient would be willing to complete the assessments when they withdraw, and record the last date the medicine was taken. Incidences of loss to follow-up and withdrawal will be recorded and reported.

### Statistical analysis

Data analysis will follow the trial’s statistical analysis plan. All data will be processed by statistical analyses with PASW Statistics 18.0 (IBM SPSS Inc., Armonk, New York, USA) and SAS 9.2 (SAS Institute Inc., Cary, USA). Two-tailed *P* values < 0.05 are considered statistically significant. Analysis will follow intention-to-treat and per-protocol subject principles. Missing data will be processed with the multiple imputation method. The baseline characteristics will be reported according to treatment groups. The EASI score will be compared between groups at 2 weeks and 4 weeks using a chi-square test, and considering the superiority comparison between the two groups via the 95% confidence interval method. The secondary outcomes NRS, IGA, Skindex-29, EQ-5D-5L, antihistamines use, CM syndrome, and tongue and pulse condition will be summarized with frequency, mean, standard deviation, median, and range.

At each time point, comparisons between the experimental group and the placebo group will be conducted using a *t* test. In order to distinguish the treatment effect and the time effect, changes from baseline in the above outcomes will be tested using repeated measure analysis of variance. Analysis of covariance or a logistic regression model will be used to adjust the central effect and covariates such as sex, age, disease course, atopic history, EASI, IGA, and BSA at the baseline. The last observation carried forward and multiple imputation method will be used to handle the missing data of continuous variables. And sensitivity analysis is used to explore the robustness of the results with different methods.

Safety will be evaluated by adverse event forms, and presented with descriptive statistics for each group. The statistics will be organized by treatment phase and post-treatment phase, as appropriate, utilizing a safety analysis set. The frequency difference of adverse events between groups will be assessed by chi-square test or Fisher’s exact test. For different AE severities, a rank-sum test will be performed to analyze the independent ordered multiple category data between the two groups.

## Discussion

This is a protocol for a multi-center, double blinded, randomized, placebo-controlled trial. This trial will be conducted in outpatient settings with experienced investigators, and participants will be recruited from 7 hospitals in different Chinese cities. The goal of this trial is to determine whether treatment with HXZQ oral liquid is of benefit to AD/eczema patients with the CM dampness pattern.

Syndrome differentiation is the fundamental principle of CM theory. Dampness pattern in CM is a syndrome differentiation type for atopic dermatitis and eczema. In China, HXZQ is commonly used for treating vomiting, diarrhea, the common cold, and insolation with the dampness pattern. Studies have reported this formula’s components’ bioactivity in enhancing cellular immunity, as well as its anti-allergy, antibacterial, and antiviral properties. For example, *Herba Pogostemonis* (Huoxiang), *Perillae* (Zisu), *Pericarpium Citri Reticulatae* (Chenpi), and *Cortex Magnoliae Officinalis* (Houpu) have been reported to have different levels of antibacterial effects. This produces antagonistic action to *Staphylococcus aureus* [21, 40–44]. Since the 1990s, reports have proliferated about HXZQ for treating skin diseases (such as eczema, urticarial, and prurigo nodularis), both internally and externally [13, 45–47]. Although many cases of HXZQ application for skin diseases have been reported, most are based on clinical experience. Few randomized trial is available to determine the benefit of this formula for skin problems caused by inflammatory reactions and/or allergies. Thus, evidence from a well-designed clinical trial is needed.

One challenge of this trial is that patient screening and primary outcome evaluation requires physicians with CM and dermatology backgrounds, or collaboration between CM physicians and dermatologists. Therefore, effective communication is needed for cooperation and support throughout the trial. Thus, we will offer a training workshop before recruitment and hire a clinical research organization to assist with data monitoring and management during the trial. Another challenge for skin disease trials is patient compliance with topical medicine. We will provide detailed instructions on the basic skin management recommended by the AD and eczema guidelines. Additionally, participants will be given moisturizers and advised to not use any topical drugs in the 24 h before the hospital visits. In this way, the primary outcome EASI can be measured more objectively. In addition, research assistants will check the data integrity of the quality of life (QoL) scale. Moreover, we will provide a separate space for participants to fill out the QoL forms out of concerns for their privacy.

At the end of this trial, we expect to know HXZQ’s efficacy in treating AD/eczema with dampness pattern in terms of both symptoms and improvement in eczematous lesions. We also want to learn whether it has a positive effect on QoL.

## Trial status

The protocol version is V1.1/20190826. The training workshop for the trial began in September 2019, and recruitment started in January 2020 and is meant to last until December 2021.

## Data Availability

The final datasets established and analyzed are not publicly available. But the corresponding author will have access to the final trial dataset and disclose contractual agreements that limit access to investigators or whom with reasonable request.

## Abbreviations

AD: Atopic dermatitis
AEs: Adverse events
AST: Alanine aminotransferase
ATL: Alanine aminotransferase
BSA: Body surface area
BUN: Blood urea nitrogen
CHM: Chinese herbal medicine
CM: Chinese medicine
EASI: Eczema Area and Severity Index
EQ-5D: EuroQol-5 Dimension
EQ-5D-5L: Five-level version of EQ-5D
HOME: Global Harmonising Outcome Measures for Eczema
HAS: Hand surface area
HXZQ: Huoxiang Zhengqi
IBRCM: Institute of Basic Research in Clinical Medicine
IGA: Investigator’s global assessment
IWRS: Interactive web response system
MID: Minimal important difference
NRS: Numerical rating scale
OTC: Over-the-counter
QoL: Quality of life
RCT: Randomized controlled trial
SD: Standard deviation
TCS: Topical corticosteroids
